# Development and evaluation of a cucumber TILLING population

**DOI:** 10.1186/1756-0500-7-846

**Published:** 2014-11-26

**Authors:** Rina Fraenkel, Irina Kovalski, Christelle Troadec, Abdelhafid Bendahmane, Rafael Perl-Treves

**Affiliations:** The Mina and Everard Goodman Faculty of Life Sciences, Bar-Ilan University, Ramat Gan, Israel; INRA-CNRS, UMR1165, Unité de Recherche en Génomique Végétale, Evry, France

**Keywords:** *Cucumis sativus*, EMS mutagenesis, Mutant screen, TILLING

## Abstract

**Background:**

Ordered collections of mutants serve as invaluable tools in biological research. TILLING (Targeting Induced Local Lesions IN Genomes) provides an efficient method to discover, in mutagenized populations, the possible phenotypes controlled by gene sequences whose function is unknown. This method can replace transgenic techniques for the functional validation of cloned genes, especially in the case of transformation-recalcitrant plants such as cucumber.

**Results:**

We report the development of a TILLING cucumber population, generated by EMS mutagenesis in the Poinsett76 genetic background. The population was evaluated by screening for morphological mutations, and a range of developmental, pigmentation and spontaneous lesion mutants were observed. Suitability for detecting single nucleotide polymorphism in selected genes has been tested by screening a sample of amplicons, with detection rate of 1 SNP in ~1 Mbp.

**Conclusion:**

The population described in this Research Note represents a useful asset in cucumber research, to be exploited for forward genetic screens and functional genomics purposes.

## Background

Reverse genetic strategies are required to attribute a function, or phenotype, to the wealth of genes whose sequences are known, but their precise role in metabolism or development is unknown. The most common reverse genetic methods in plants involve T-DNA insertions or RNAi silencing, however, both methods require an efficient plant transformation platform and long term efforts to generate large arrays of transgenic lines. TILLING (Targeting Induced Local Lesions IN Genomes) is a relatively inexpensive, non-transgenic technique for interrogating the function of a given sequence; it is suitable for any species, regardless of genome size [1–3]. To generate a saturated TILLING population, wild-type seeds are typically treated with EMS (ethyl methanesulfonate), a mutagen that saturates the genome with point mutations, mostly G/C to A/T transitions [4]. The treated seeds are germinated, and the resulting M_1_ plants self pollinated to collect M_2_ seeds. DNA samples from mutagenized seed pools are screened by PCR amplification of the gene of interest, followed by digestion with an endonuclease that cleaves at mismatched sites. If a particular M_2_ family carries a point mutation in the amplified fragment, heteroduplex DNA carrying a mismatch at the mutated site will form during replication of mutant and wild-type alleles present together in the particular family-sample. Genetic analysis of the segregating family, *i.e.,* sequencing individuals from the suspect family and their phenotypic inspection, allows the discovery of mutant phenotypes and sheds light on the gene’s function.

In a TILLING population, mutations are spread randomly in the genome; a spectrum of mutated alleles, including weak ones that are desirable for studying essential genes, can be obtained, and can even be used to infer structure-function relations of the encoded protein [5, 6]. The method has been refined by Bendahmane and co-workers for different crops, including *Cucumis melo*, the melon [7, 8]; see also [9], pea [10] and tomato [5]. The use of an Arabidopsis endonuclease, ENDO-1, rather than the commonly used CEL-1 nuclease from celery, rendered the screening of molecular mismatches particularly efficient [7, 11, 12].

Cucumber *(Cucumis sativus)* is a species of economic importance, whose genome has been sequenced recently [13–15]. However, being transformation-recalcitrant, functional genomics studies in cucumber have lagged behind. Here we report on a TILLING population that could enhance the cucumber genomic toolkit for future research. Studying a sample of the population, we show the range of morphological mutations that is seen, and demonstrate the occurrence of nucleotide substitutions in a few gene fragments.

## Results and discussion

### Mutagenesis and multiplication

**Figure 1:**
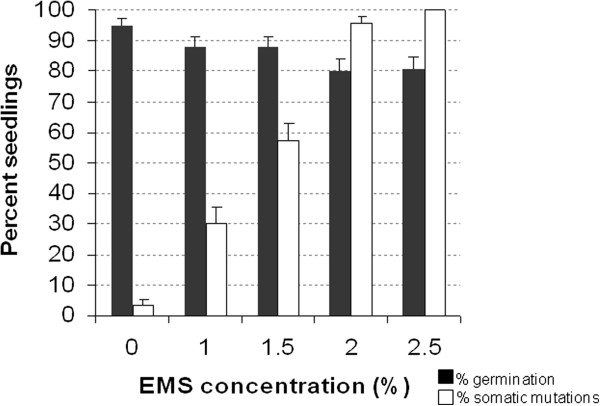
**Calibration of EMS treatment**. Aliquots of 100 seeds were treated with 0, 1, 1.5, 2 and 2.5% EMS and sown in germination trays. Percent germination was recorded after 9 and 16 days, and seedlings exhibiting somatic mutations in the cotyledons, first and second true leaf were counted and expressed as percent of the fully germinated seedlings. Mutations included smaller or distorted cotyledons or leaves, as well as dark–light leaf patterns. Standard errors of the percentage of the final germination or mutated seedlings rates was computed as 100× [b × (1-b)/n]^0.5^, where b is the proportion of a given class, and n – the total number of plants [17].

To select the optimal EMS concentration that is likely to produce many mutations but will not heavily affect germination and fertility, we performed two calibration experiments, in which seeds of cucumber ‘Poinsett76’ were exposed to 0–2.5% EMS. Figure 1 displays the results of an experiment, in which 100 M_0_-seed aliquots were treated with five concentrations (0, 1, 1.5, 2, 2.5% EMS, respectively). Germination rates were recorded and ranged between 95% (untreated seeds) and 80%, being moderately affected by the mutagen. We also recorded the rate of seedlings exhibiting somatic mutations, *i.e*., leaf distortion and mosaicism (dark/light variegation) on the first and second true leaves. Such rates increased gradually from 3% total ratio of apparent abnormalities in untreated seedlings, to 97% and 100% in the 2% and 2.5% treatments, respectively. We selected 1.5%-2% EMS as optimal concentrations that mildly affected germination but had a substantial proportion of visible somatic damage. Sub-samples of these plants were grown further and we noted that in most plants the new leaves that developed were normal, and the mosaic symptoms recorded in the first leaves did not extend to subsequent leaves. Apparently, the somatic mutations did not affect the shoot apical meristem but only the first and second leaf-primordia in the mutagenized seed; only a small proportion displayed persistent mutations at the M_1_ generation. A similar recovery of melon M_1_ plants from growth inhibition following 1-2% EMS mutagenesis was reported by Dahmani-Mardas et al. [8]. For pea, however, a lower 0.25% concentration (20 mM) was selected [10], with less than 30% of the plants setting seeds at higher concentrations; in Arabidopsis, high mutation rates were achieved by 0.25-0.5% EMS [16] and the authors suggested that DNA repair mechanisms could be responsible for inter-specific differences in mutation yields.

Thirty seedlings from the 1%, 1.5% and 2% treatments were transferred to the greenhouse for further growth and fertility assessment. We concluded that fertility following self pollination was similarly good at all three EMS concentrations, with 87% of the plants treated with either 1.5% or 2% mutagen producing >100 seeds. This agrees with Dahmani-Mardas et al. [8] who reported that 3% EMS, but not 1-2%, significantly affected melon fertility.

To produce the TILLING population, we applied 1.5 or 2% EMS to Poinsett76 cucumber seeds in three different batches. A total of 1200 M_1_ plants were grown to full maturity in a farmer’s net-house in Netiv Ha’asara, Israel Southern coastal region, and in Bar-Ilan University net-house, and self-pollinated to collect M_2_ seeds. This resulted in ~1000 M_2_ families with adequate yield of 50–300 seeds. Six seedlings per M_2_ family were germinated, and a mixed DNA sample from four of them was prepared. The first 768 families were arranged in 96 well plate wells (see Methods) and used to evaluate the population.

### Phenotypic evaluation of the population

**Figure 2:**
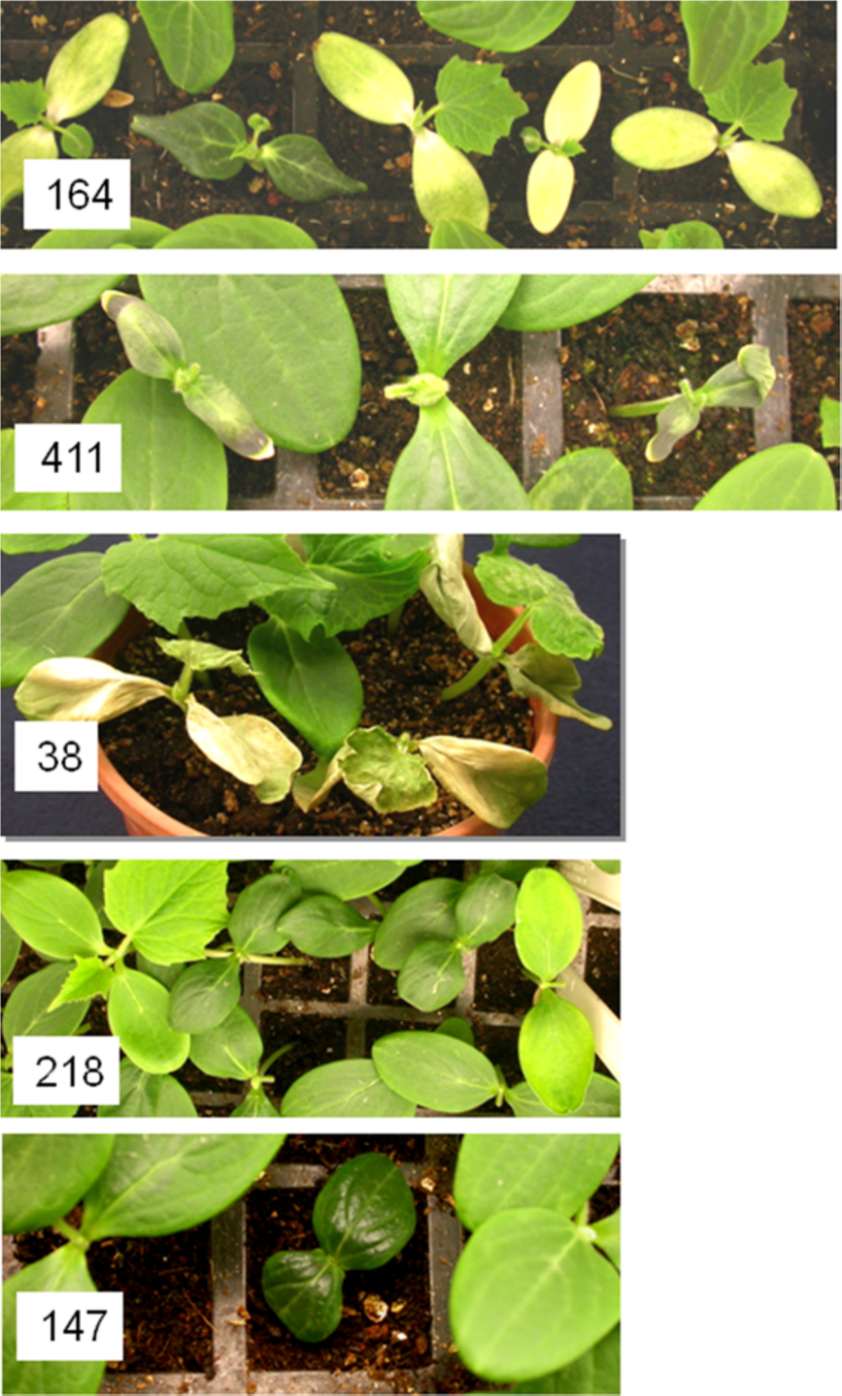
**Selection of morphological mutations segregating in M**_**2**_ **mutated families at the seedling stage.** Family 164: albino cotyledons, green leaf. Another mutation for pointed cotyledons seems to segregate as well. Family 411: dark, seemingly anthocyanin-enriched, narrow cotyledons. Family 38: spontaneous necrosis of cotyledons and leaf. Family 218: yellow-light green pigmentation. Family 147: dwarf phenotype, small dark cotyledons.

We utilized the M_2_ seedling samples (6 individuals per family) grown for DNA extraction to record mutant phenotypes that were observable at the cotyledon and first leaf stages. Whereas we cannot exclude that some phenotypes could be due to environmental variation or seedling physiology – especially differences in size or germination ability - in many cases, the phenotype was apparent in two individuals of the same family, demonstrating the likely heritability of the putative mutation. Table 1 summarizes the phenotypes that we recorded and Figure 2 provides several examples. The most frequent seedling phenotypes included post-germination lethality, dwarfism, and spontaneous lesions in the cotyledons. The latter class was unlikely to result from response to pathogens in the growth chamber, as it appeared sporadically, often with two seedlings per family showing the lesions while the rest of the sample and the adjacent seedlings in the tray lacked them. Such mutations could identify genes involved in disease-resistance signaling. Mutants with distorted cotyledons and/or leaves, albino cotyledons and yellow or mosaic-yellow leaves were also apparent, as well as taller seedlings, dark-narrow and fused cotyledons. In total, 10% of the families exhibited a morphological alteration.

**Table 1:** **Major mutant phenotypes recovered by visual screening of the TILLING population at the seedling stage**

Phenotype	No. of families displaying phenotype	Description	Representative family
Seedling lethality	19	Seedling dies, or fails to develop a root after germination	144, 157
Dwarf	10	Cotyledons smaller, short hypocotyl	422
Necrotic lesions	15	Spontaneous necrotic spots appear on cotyledons	38
Albino	3	White cotyledons	164
Yellow leaf	7	True leaves pale-green or yellow, or mosaic green and yellow	218
Tall seedlings	5	Hypocotyl >2 cm taller than wild type	180
Glabrous	1	Cotyledons lack trichomes	16
Non-serrated leaf	3	Leaf edge appears smooth	189
Dark, narrow cotyledon	1	Dark green, narrow cotyledons	411
Fused cotyledons	1	Cotyledons fused together	714
Small leaf	7	True leaf very small	17
Distorted leaf/cotyledon	5	Irregular organ shape	160, 213
Total mutants/ families screened	77/768		

Six seedlings per M_2_ family were sown in trays and inspected at the cotyledon-first true leaf stage. The number of families and examples of specific families that segregate for a given phenotype-class are indicated. About 10% of the families exhibited morphological alterations at the seedling stage.

**Figure 3:**
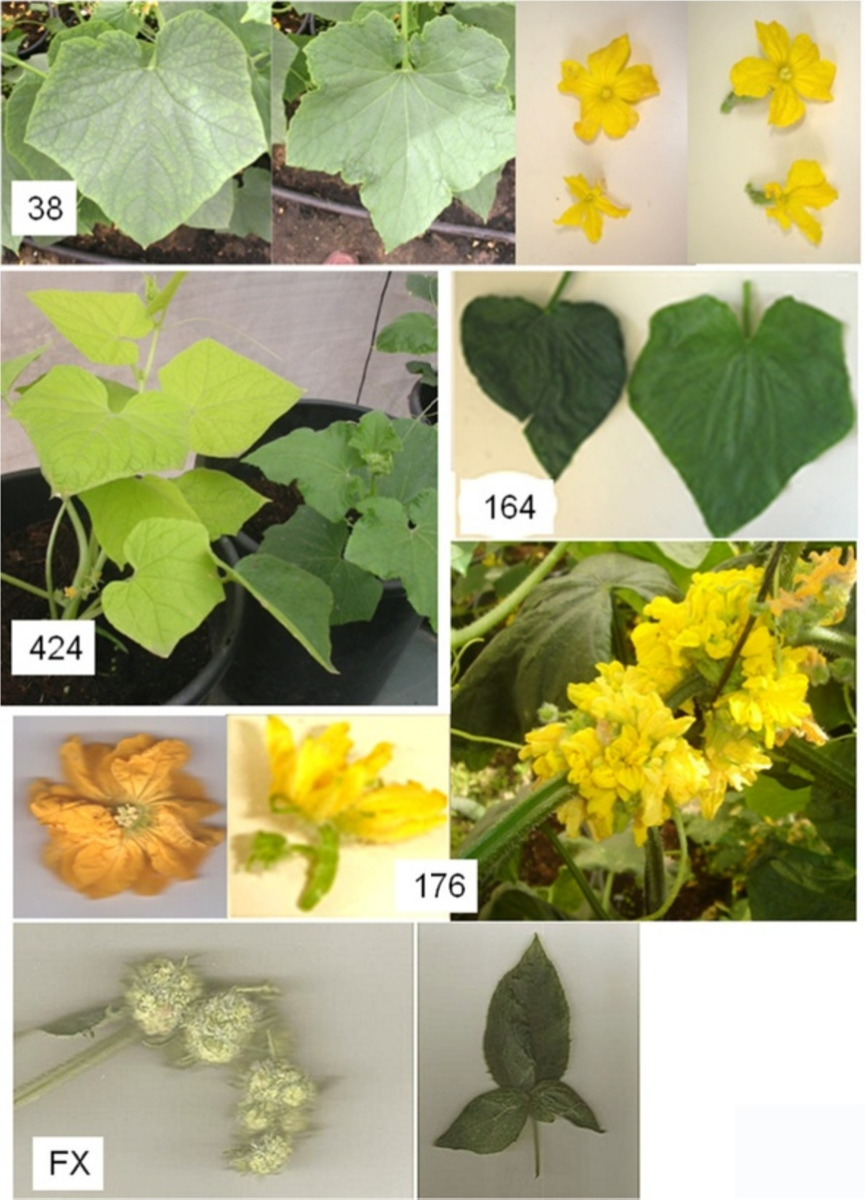
**Selection of morphological mutants at the mature plant stage.** Family 38: deeper-lobed leaf (right, compared to wild-type leaf on the left), and smaller male and female flowers (bottom, compared to wt flowers on top). Family 424: pale green plant. Family 164: darker leaf with shallow lobes (left, compared to wt leaf on the right). Family 176: fasciated, sessile inflorescences with reiterated organs, multiple petalled flowers and a branched ovary. Family FX: “cauliflower” mutant with arrested-development, reiterating, inflorescence with dense trichomes and a lanceolate leaf.

Several seedlings that exhibited prominent abnormalities were grown to maturity, and outstanding phenotypes could be seen among the mature plants as well. These included persistent virescent or yellow-leaf character (Family 424), a fasciated plant with floral organ abnormalities and organ-fusions (Family 176), and more; Figure 3 provides a few examples. This demonstrates that the population is a rich source of morphological and developmental mutations that could be tapped using forward-genetic schemes. Since each M_1_ plant and the descendant M_2_ family harbors multiple point mutations, genetic analysis will be required to discern them and correlate a specific mutant phenotype with a single molecular event.

### Molecular evaluation of test-genes

**Figure 4:**
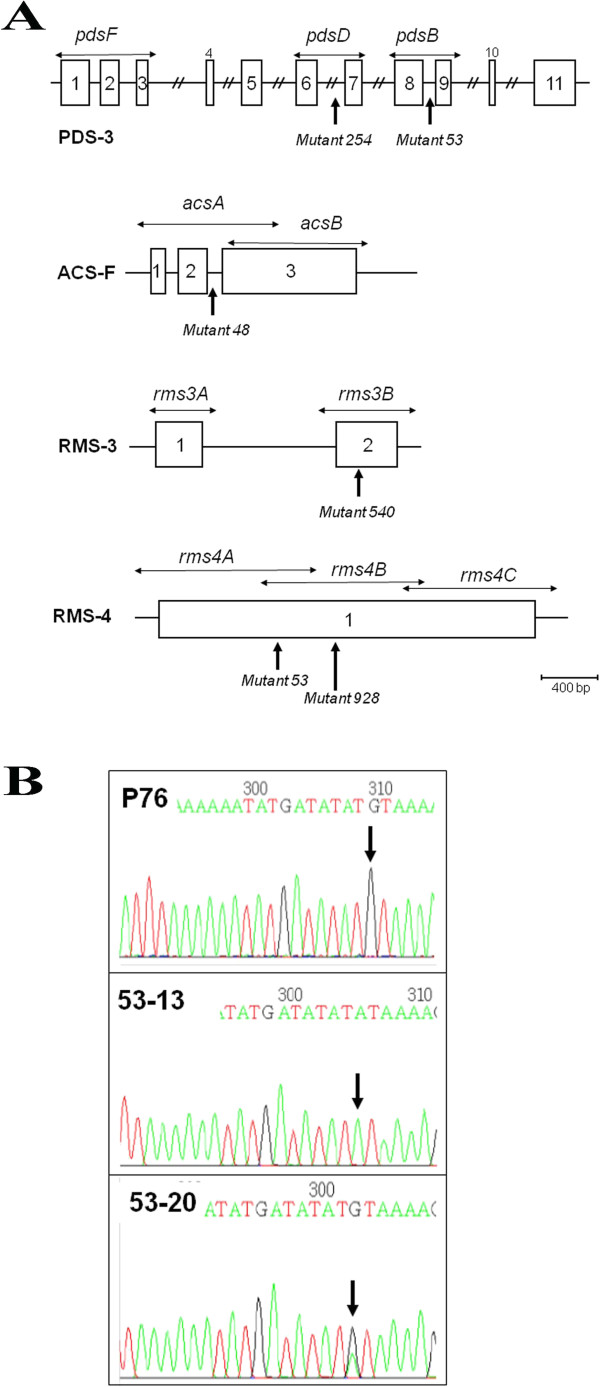
**Screen for nucleotide substitutions in selected gene fragments. A**. Gene model and amplification schemes of the cucumber *phytoene desaturase-3* gene, *Female* ACC synthase, *ramosus-3* and *ramosus-4* homologous genes. The approximate positions of the amplicons screened by TILLING are indicated, each delimited by two pairs of nested primers (arrows indicate the internal amplicons). The mutations that were verified by sequencing (from families 53, 254, 48, 540, 928) are indicated; in family 53, two independent point mutations were recovered in two of the genes, respectively. **B**. Chromatogram of the C to T mutation (read by a reverse primer as G to A), discovered in the *PDS-3* gene, in family 53. Top: wild type sequence in cultivar Poinsett76. Below: plant 53–13 is homozygous for the mutation, plant 53–20 is heterozygous.

To check whether we could interrogate our population with a given gene sequence and recover point mutations in selected gene fragments, we chose six genes that yielded visible phenotypes when mutated in other plants. Primers were designed to amplify the more conserved parts of the coding sequence; in some cases, intronic sequences were also included. Figure 4A depicts the amplification scheme of four genes, for which point mutations were recovered. Table 2 specifies the primers that were used.

**Table 2:** **Primer pairs used to generate amplicons for the TILLING screen of six genes, as detailed in the methods**

Gene	Amplicon	External primers	Internal primers (M13 tag bold)
*PDS-3*	pdsB	Pds3for1, CACAGATGACATTCTTCCCAAT	pds3for3, **CACGACGTTGTAAAACGAC**TCTAACATACCCATAGG
		Pds3rev2, CCTAGTTCTACCCTTTGTTCTTGG	pds3rev4, **ATAACAATTTCACACAGG**GTTTCATGCTGGCTGCC
	pdsD	Pds3for5, GGAAATTTGTCTCAACATGTGTGC	Pds3for7, **CACGACGTTGTAAAACGAC**TCTCCAAACTAGTGAC
		Pds3rev6, CTTGTGCCACATGGCTAGAATAG	Pds3rev8, **GATAACAATTTCACACAGG**CTGCCGGTGATGCTGG
	pdsF	Pds3for9, AAGGGGCTCGACTGTTCAGAAA	Pds3for11, **CACGACGTTGTAAAACGAC**CACTAGAAGACTCAGC
		Pds3rev10, CTGGTAGTGATTCTCGGTTTCA	Pds3rev12, **GATAACAATTTCACACAGG**CATCCACCGAAGTAGA
*Female*	acsA	AcsFor1, GAACTATCTACCATATTCCAACC	AcsFor2, **CACGACGTTGTAAAACGAC**GTACCTATATACCTCACCTCAACAT
		AcsRev3, CACCAACTCGAAAACCTGGGAGCC	AcsRev1, **GGATAACAATTTCACACAGG**CCTCGTCTTCGTTACTCCTCTCCT
	acsB	AcsFor5, TTGATAGAGATTTGAAATGGAGA	AcsFfor6, **CACGACGTTGTAAAACGAC**CCGGAGTTGAGATTGTGCCAATTC
		AcsRev7F, CCGAGTGCACTTTTCTTTTTC	AcsRev5, **GGATAACAATTTCACACAGG**ATTCCCCCAAATATGGATGATG
*RMS-3*	rms3A	Rms3for1, GTGTCACCGTGCATGCAATTGCCG	Rms3for2, GTACACTTCAAATCATAAACGGCTG
		Rms3Rev2, TTCATCTCTCAGTTTTCCTACCTAAT	Rms3Rev1, GGGTTACAAACGCTGGCCTTC
	rms3B	Rms3for6, ATTGCAAACATAGCCATCAAAATC	Rms3for7, GTCAAAATTATCATTTCTACGCAGG
		Rms3rev6, CAAGTAAAAACACAGCTCTCAACCT	Rms3Rev5, GAGTGGATGCTATTCCTTTTCGATG
*RMS-4*	rms4A	Rms4for5, CTCTCTCCGTTGCTAAGACAAACCC	Rms4for1, TCCGATTACTGTATCTTCCTGCTCG
		Rms4rev6, CAAACTCACCATTGTTCTCAAACCC	Rms4rev1, CGACAGCGATTCAAGCCCTTGACAA
	rms4B	Rms4For4, TTCTCGTGGCCAATCCTCTGAC	Rms4for2, CCATTACCGAGGCTTGCCCTAACCT
		Rms4rev5, GCCCCACAAATCATTACCACTGCAT	Rms4rev2, GCCCCACAAATCATTACCACTGCAT
	rmsC	Rms4G3for7, GGATGGAAACTATGGTGGATATA	Rms4for3, CCAGGTACTCCACCGACGCTGATTG
		Rms4G3rev7, CCCATGAAAGATTGTGAAATCACA	Rms4rev3, GTGATACAGCTAATCTCAAAGTAAC
*Cum-1*	Cum1B	Cum1for1, GCTCTTTTCCTCATCAGGTTAGTG	Cum1for3, **ACGACGTTGTAAAACGAC**TCTCCTGCTCATTCCAC
		Cum1rev2, GTATACACCAAACTGAGAACCAG	Cum1rev4, **GATAACAATTTCACACAGG**TACAACCCAGATTCCC
	Cum1D	Cum1for5, GATATCAATTAAACCATGCGGGC	Cum1for7, **CACGACGTTGTAAAACGAC**TGGTATGAAATGGGGG
		Cum1rev6, GATTATCGGTTTCATCTCCATGG	Cum1rev8, **GATAACAATTTCACACAGG**CCCAATTCAGACCTTC
*SP*	spB	SPfor1, GGACAGCACAAGAAAAGGTCAC	SPfor2, **CACGACGTTGTAAAACGAC**TGCCTCTCTCTCTGCT
		SPrev1, CACATCATTTCTTGCCAATTGTC	SPrev2, **GATAACAATTTCACACAGG**GGGCATCTTTTGCAAC
	spC	SPfor1, GGACAGCACAAGAAAAGGTCAC	SPfor3, **CACGACGTTGTAAAACGAC**CAACCAATTCCCAAAC
		SPrev1, CACATCATTTCTTGCCAATTGTC	SPrev3, **GATAACAATTTCACACAGG**CACTCTCTCTCACCAG

The bold portion of the internal primers corresponds to the M13 tail that binds a third pair of universal primers that were fluorescently labeled. The corresponding internal primers of *Rms-3* and *Rms-4* were directly labeled.

Cucumber *phytoene desaturase-3* (*Pds-3*), gene accession number Csa002881, encodes a carotene biosynthetic enzyme. Inactive alleles could give rise to an albino phenotype [18]. We screened three amplicons and identified four mutated families, two in amplicon B and two in D. We sequenced and verified the mutations in families 53 and 254, and each carried a different C-to-T substitution. In family 254, three plants were heterozygous and two had the wild type allele; in family 53, two were wild-type, two homozygous for the mutation and four were heterozygous (Table 3, Figure 4). All four mutations mapped to introns present in the amplicons, and no mutant phenotypes were observed. This underscores the importance of targeting exons and avoiding introns when performing a TILLING screen.

**Table 3:** **Nucleotide substitutions recovered in query genes by TILLING screen**

Gene	Amplicon	Amplicon size, bp	% GC content	% Exons	Mutated family	SNP	Diagnostic CAPS	Location/substitution	M3 Progeny analyzed: ***WT/WT : Heteroz.: mut/mut***
*Phytoene desaturase-3 (PDS-3),* Csa002881	pdsB	622	36.5	55	53	C5310T		Intron 8	*2 : 4 : 2*
					188	nd		Intron 8	*nd*
	pdsD	1198	34.5	23	254	C3997T	-	Intron 6	*2 : 3 : 0*
					70	nd		Intron 6	*nd*
*ACC synthase (F),* Csa012150	acsA	1111	39	70	none	-	-	-	-
	acsB	1025	41	96	48	C594T	*Dra*I	Intron 2	*4 : 9 : 5*
*Ramosus-3 (RMS-3),* Csa010158	Rms3A	513	47	72	none	-	-	-	-
	Rms3B	639	43	70	540	G1376A	-	Gln191Gln	*3 : 4 : 0*
*Ramosus-4 (RMS-4),* Csa003326	Rms4A	1222	51	86	53	C767T	-	Thr256Ile	*3 : 10 : 3*
	Rms4B	929	46	100	928	C1258T	*Sac*I / *Xba*I	Leu420Leu	*7 : 7 : 0*
	Rms4C	798	45	84	none	-	-	-	-
*Cucumber MADS1 (Cum1)*, Csa000681	Cum1B	575	35	41	none	-	-	-	-
	Cum1D	702	34	30	none	-	-	-	-
*Self-pruning (sp),* Csa010707	SpB	704	29.8	39	none	-	-	-	-
Total		10,766	39.7	63	8				

Genes are indicated by name and by accession numbers (cucumber genome project, http://cucumber.genomics.org.cn). Amplicon size (calculated between internal primer pairs) is shown, as well as the GC composition and the ratio of coding sequence (exons) to total amplicon length. Mutated position is determined according to the genomic sequence, from the start codon (ATG), and its location, either in an intron or in the protein coding-sequence, is indicated. Nd – non determined. CAPS marker in *ACC synthase* gene: the wild type amplicon is digested by *Dra*I, mutant amplicon is uncut. CAPS marker in *RMS4*: wild type amplicon is digested by *Sac*I (and also *Xba*I), no restriction in the mutant. A small number of M_2_ progeny was genotyped to demonstrate inheritance of the nucleotide substitution. The total sequence screened (10,766 bp) was calculated by summing up all the internal amplicons, and detracting the overlapping regions found between the *Acs (F)* and *Rms-4* amplicons (see Figure 4).

The *Female* sex-determining gene encodes an ACC synthase enzyme that controls the differentiation of female flowers and abolishes male flowers [19]. We detected a single nucleotide substitution in our screen that segregated in the expected 1:2:1 ratio, and mapped it to intron 2 of the gene (Table 3, Figure 4A). As expected from its intronic location, the mutation had no phenotypic effect.

*Ramosus-3* and *Ramosus-4* are genes that affect apical dominance and were extensively studied in pea, as well as other plant species [20]; *rms* mutants often show extensive branching. We designed and screened amplicons for each gene, and recovered one mutant in *rms-3* and two in *rms-4*. All three substitutions mapped to the coding sequence (Table 3, Figure 4A). Two of them involved silent mutations and no phenotype was observable. The third mutation, in the single large exon of the gene (Family 53) caused a threonine-to-leucine substitution. In the M_2_ generation, we did not observe progeny with increased branching, or other phenotype that we could associate with certainty with the Thr256Ile substitution. We have analyzed the respective protein sequence using SIFT (Sorting Intolerant From Tolerant, [21]) and found that substituting Thr to Ile at this position is predicted to be tolerated and thus, unlikely to affect the phenotype. In addition to the above four genes, we screened also a cucumber homolog of the tomato *self-pruning* gene for determinate growth habit (cucumber *sp*, Csa010707) and a MADS box homolog, *CUM-1* (Csa00068; Tables 2 and 3), but no mutations were recovered for these two genes.

We have recovered, by screening six genes, in a 768 families-sample of the population (of the ~1000 presently available), a total of 8 mutants, 6 of which were confirmed by sequencing the M_2_ progeny; the other two were estimated to be in the intron, judging from their endonuclease product sizes, and were not analyzed further. The mutations were recovered by screening 13 amplicons that totaled 10,766 nucleotides (Table 3). The average mutation frequency in the collection is, therefore, 8 mutations per 10,766 × 768 bp of sequence screened across the population, *i.e.,* 1 in 1.03 Mbp. If we ignore amplicon borders, where endonuclease digestion would pass undetected, frequency would be 1 mutation in 859 kb. Such density is fair, albeit lower than the density observed by for pea (1/200 kbp, [10]), *Arabidopsis* (1/300 kbp, [16] and melon (1/573 bp, [8]); it is similar to the observed rate in barley (1/1 Mbp, [22]) and allows for Reverse Genetics screening of the population. In a parallel study, another TILLING population has been prepared in the cucumber ‘Beit-Alpha’ background, and a similar mutation density (1/1.14 Mbp) was recovered [23].

In the amplicons that we screened, we could compare SNP rates in exons (comprising 60% of the amplicons) *vs* introns (40%). Of the eight mutations, five were located in introns and three in exons. Of the six sequenced mutations, five were C to T, and one was a G to A transition, which are the two common products of EMS mutagenesis [16]. The ratio of intronic mutations exceeded the ratio of exonic ones by a factor of 2.5 (= 60/40 × 5/3). Moreover, two of three exonic mutations were silent; it has been estimated that about half of the non silent (missense) mutations are likely to affect protein activity [24]. Thus, if one wishes to recover mutated alleles with a higher probability of affecting the phenotype, population size should be increased to ~3000 plants and inclusion of introns in the amplicons screened should be minimized, although, when exons are small, introns are hard to avoid. The present SNP sampling was not aimed at recovering mutant phenotypes, the goal being a mere assessment of DNA-level mutation rates.

By sequencing a small sample of progeny of mutated families in the M_2_ generation we confirmed the Mendelian inheritance of the nucleotide substitutions. In two cases we facilitated screening by developing a diagnostic CAPS marker. In three cases we did not recover the homozygous-mutant class (Table 3). Since we did not expect the respective mutations to be lethal (none of them affects the protein product of the gene), this segregation could be due to the small sample of progeny, or to pollination of a heterozygous mutant plant with wild-type pollen; this, in turn, could result from out-crossing with a neighbor plant, or from a chimeric plant composition following seed mutagenesis. In such cases, self pollinating of heterozygous individuals can readily provide the desired homozygous mutants.

## Conclusions

A cucumber mutant population in the Poinsett76 background was successfully constructed using 1.5-2% EMS treatment. It provides a rich source for morphological mutant phenotypes that can be recovered by forward genetic screens. It can also be efficiently screened by the reverse genetics TILLING approach, to recover mutations in target genes. With the entire cucumber genome sequence available, our study thus provides a valuable tool for functional genomics in cucumber. Data and seeds for collaborative research can be obtained by agreement from the corresponding author.

## Methods

### Preparation of the TILLING population

Poinsett76 cucumber seeds were incubated at room temperature for 15 hrs, in 3 volumes of freshly-prepared EMS solution (Sigma M0880), in 0.2 M phosphate buffer (pH = 7), with gentle stirring. After extensive washing (5 × 30 min in tap water at room temperature with stirring), seeds were sown in “Speedling” germination trays and transplanted in 10 L pots in the greenhouse at the 2–3 leaf stage. Plants were grown under standard agronomic conditions, self-pollinated, and M_2_ seeds were harvested (50–300 per plant). From each M_2_ family, six seeds were sown, and after two weeks, 4 young leaf discs were sampled from four individuals of the family (8 mm diameter, one disc per individual) and pooled for DNA extraction by the CTAB method [25]. Uniform DNA concentrations were obtained by suspending in 200 μl water and samples were kept at -80°C. Individual family-samples of the first 768 families were arrayed in eight 96 well plates, and DNA aliquots were diluted 10 times (to obtain a 400 μl volume, ~10 ng DNA/μl) and kept at -20°C. DNA pools were prepared (8 families /pool) and arrayed in a single 96-well pooling-plate representing 768 families. Such sampling strategy was possible due to the highly sensitive TILLING screen that allows detection of mutant alleles in the eight-family x four individuals mixed sample.

### Target gene amplification and screening

Amplification included two steps of nested PCR. The first involved a pair of external unlabeled primers, 22–25 nt-long, 40-50% recommended GC content, planned to generate a fragment up to 1.2 kb in size. Primers were reacted with 2 μL of the pooled genomic DNA (~20 ng) for 30 PCR cycles. The second round of PCR was performed using 1 μL of the first reaction products as template, with two pairs of internal primers present together in the same reaction. The first primer pair in the second round comprised a 3’ region that specifically binds the PCR products of the first round (having a 40-50% recommended GC content), while the 5’ region of the primer represents a universal M13 sequence “tail”, resulting in a 35 nt-long primer. The last primer pair (M13F700, 5’ CACGACGTTGTAAAACGAC and M13R800, 5’ GGATAACATTTCACACAGG) is fluorescently marked (IRDye™ 700 and IRDye™ 800) and matches the M13 sequence tags of the second primer pair. For the *rms-3* and *rms-4* genes, the second pair of primers was fluorescently labeled and M13-labeled primers were not required. The reaction included 35 cycles, the first 10 performed at the recommended annealing temperature of the specific primer pair, then 25 cycles at a lower annealing temperature, 50°C, required for the universal primers [3, 12]. Finally, heteroduplex molecules were formed by a temperature gradient of 94°C to 8°C (-0.1°C/sec).

The products were digested for 20 min at 45°C with EndoI endonuclease and separated on a polyacrylamide sequencing gel using a LICOR 4300 machine. The pooled 96 DNA samples were screened, and positive family-pools displaying amplicon digestion were deconvoluted to identify the positive M_2_ family within the pool. The family’s DNA sample (that had been prepared by mixing four individual progeny) was sequenced, to look for a mixed peak, indicating a mutation at a position predicted by the endonuclease digestion pattern. A few progeny of the family were sown and their amplicon sequenced, to confirm the mutation and correlate it with a possible phenotype. In a few cases, the mutation generated a restriction site polymorphism; in that case, genotyping was performed by restriction digestion of the PCR product, followed by agarose gel electrophoresis and ethidium bromide staining.

## Abbreviations

CAPS: Cleaved amplified polymorphic sequence
EMS: Ethyl methanesulfonate
PCR: Polymerase chain reaction
SNP: Single nucleotide polymorphism
TILLING: Targeting Induced Local Lesions IN Genomes.
